# Development of a LeNet-5 Gas Identification CNN Structure for Electronic Noses

**DOI:** 10.3390/s19010217

**Published:** 2019-01-08

**Authors:** Guangfen Wei, Gang Li, Jie Zhao, Aixiang He

**Affiliations:** 1 School of Information & Electronic Engineering, Shandong Technology and Business University, Yantai 264005, China; sdgshax@126.com; 2 Key Laboratory of Sensing Technology and Control in Universities of Shandong, Shandong Technology and Business University, Yantai 264005, China; 3 School of Computer Science & Technology, Shandong Technology and Business University, Yantai 264005, China; 13054556909@163.com (G.L.); guapi0208@163.com (J.Z.)

**Keywords:** gas identification, electronic nose, pattern recognition, convolutional neural network

## Abstract

A new LeNet-5 gas identification convolutional neural network structure for electronic noses is proposed and developed in this paper. Inspired by the tremendous achievements made by convolutional neural networks in the field of computer vision, the LeNet-5 was adopted and improved for a 12-sensor array based electronic nose system. Response data of the electronic nose to different concentrations of CO, CH_4_ and their mixtures were acquired by an automated gas distribution and test system. By adjusting the parameters of the CNN structure, the gas LeNet-5 was improved to recognize the three categories of CO, CH_4_ and their mixtures omitting the concentration influences. The final gas identification accuracy rate reached 98.67% with the unused data as test set by the improved gas LeNet-5. Comparison with results of Multiple Layer Perceptron neural networks and Probabilistic Neural Network verifies the improvement of recognition rate while with the same level of time cost, which proved the effectiveness of the proposed approach.

## 1. Introduction

Electronic nose (EN) refers to a system that simulates the olfactory system of humans and other mammals in structures and functions [1] to achieve the qualitative and quantitative analysis of gases or odors, which is also called the artificial system of olfaction. At present, ENs have been widely studied and applied in medical diagnosis [2], food quality testing [3], environmental monitoring [4], etc. Gas identification method plays a great important role in EN systems with a settled sensor array, which makes the study on gas identification approaches a research hot spot in gas detecting areas.

Lots of gas classification and identification methods based on pattern recognition technology have been studied, such as the Principal Component Analysis (PCA), Linear Discriminant Analysis (LDA), k-Nearest Neighbor (k-NN), and Artificial Neural Networks (ANNs). PCA is a generally used data dimension reduction and clustering method. LDA is a general linear statistical approach. Both PCA and LDA have been the traditional gas sensor array data processing methods. In Ref. [5], k-NN has been proved to be a simple and effective method for clustering. ANNs can not only solve complex nonlinear mapping relationships, but also improve the accuracy of classifications, which have shown good results in the qualitative and quantitative identification of harmful gases [6]. In ANNs, Multiple Layer Perception (MLP) is widely adopted to the study of gas classification [7]. All these proposed gas classification approaches can be concluded into shallow models [8] from the view of machine learning structures.

However, with the development of artificial intelligence, deep learning techniques have attracted a large amount of interest and shown better results than shallow models. Several deep learning models have been studied in the gas identification area. A Digital Multi-layer Neural Network (DMNN) was proposed in Ref. [9], which can achieve over 93% classification performance. In Ref. [10], Langkvist et al. put forward a deep-restricted Boltzmann machine (RBM) combined with an electronic nose to identify bacteria in blood. In Ref. [11], gas identification research using a deep network is also introduced (deep Boltzmann Machine (DBM) and Sparse Auto-Encoder (SAE)), and the accuracy of the experimental results is higher than that of the traditional shallow model. In these articles, RBM, DBM and SAE are all unsupervised learning techniques that can learn higher-order features from large amounts of unlabeled data. As a supervised deep learning method, Convolutional Neural Network (CNN) shows attractive development in AI. A Deep Convolutional Neural Network (DCNN) was used to classify gases in Ref. [12]. The authors designed a network with six convolutional blocks, a pooling layer and a fully connected layer to increase the depth of learning. Their final experimental result has an accuracy of 95.2%, which is higher than SVM and MLP. All these works show good prospective applications of deep learning methods in gas identification fields.

Recently, many typical and widely adopted CNN models have been proposed, such as LeNet-5 [13], AlexNet [14] and GoogLeNet [15], which have been successfully applied in handwritten character recognition [16], face detection [17], pedestrian detection [18] and robot navigation [19] areas. Due to its high recognition rate and fast implementation speed, CNN continues to make efforts in many directions and breakthroughs.

Enlightened by the above applications and developments of deep learning techniques, this paper pays attention to a detailed study of a CNN-based gas classification method for ENs. The general LeNet-5 structure is improved and developed for EN gas identification with less convolution blocks but higher computation speed. The feasibility of the network structure is verified by experiments. Section 2 describes the EN system and experimental setup; Section 3 describes the traditional LeNet5 structure; Section 4 describes the algorithms; Section 5 describes the Gas CNN algorithm. Section 6 analyzes the experimental results and proposes the improved LeNet-5 structure for ENs; Section 7 presents the conclusions.

## 2. The EN System

### 2.1. The EN Frame

**Figure 1:**
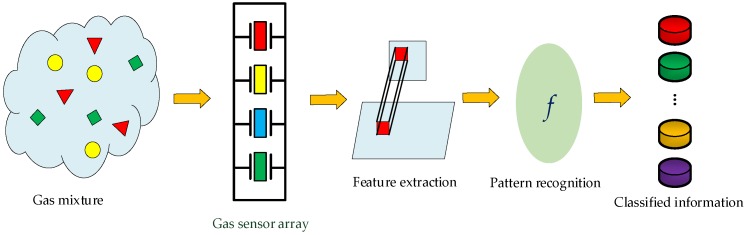
The frame diagram of EN.

Generally, an EN is composed of a gas sensor array and a gas quantification/qualification unit. Figure 1 shows a typical frame diagram of an EN. The sensor array consists of a certain number of gas sensors, which plays the sensing role for gas mixtures. The sensor array responses are transferred and conditioned by the designed interface circuit and then acquired by a DAQ board [20]. The characteristics of the response signals are then extracted out as the useful features, and the extracted features are continuously put into a pattern recognition unit for classification and quantification. Finally, information of the type and concentration of the gas components in mixtures can be obtained.

### 2.2. The EN System and Its Test Equipment

In this paper, 12 commercial metal oxide gas sensors from Figaro Ltd. (Minami, Japan) were selected to constitute the sensor array. Table 1 shows the part numbers of the sensors used and their corresponding channel numbers. These sensors are widely responsive to general flammable or explosive gases in the environment. The array was placed into a stainless steel chamber with volume of 138 mL, which is 11.5 cm × 4 cm × 3 cm.

**Figure 2:**
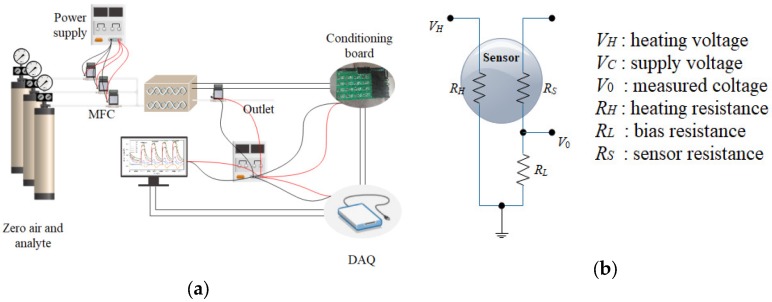
(**a**) The improved system of the EN and the automated test system; (**b**) The measurement circuit.

The sensors were operated at their recommended working temperatures, and are heated up by a RH with a fixed heating voltage of 5 V. The variance of sensor resistance is obtained through a voltage divider circuit with a bias resister *R_L_*, while *V*_0_ is the output of the sensor and acquired by a DAQ board. Figure 2a shows the diagram of the gas distribution and EN detection equipment, and Figure 2b shows the measurement circuit of gas sensors.

The NI USB6342 multi-functional DAQ board is adopted as the data acquisition device with a USB interface to facilitate connection with the PC control terminal. High-precision Mass Flow Controllers (MFCs, Alicat Ltd., Tucson, AZ, USA) were selected for airflow control, which was controlled by the PC through the RS-232 interface protocol. The conditioning board was designed between the DAQ and the sensors in chamber, for the purpose of adjusting the strength of the output signals. The analytes to be measured were diluted by zero air, and their concentrations were controlled through ratios of flow rate of each MFC. The assembled analytes are injected into the test chamber with stable total flow rate. Both the data acquisition and gas distribution were controlled by PC via a LabVIEW program.

### 2.3. Data Measurement

The analytes in this experiment are two flammable and explosive gases: CH_4_ and CO. Based on their harmful level and general industrial needs, the concentrations of CH_4_ are set at 500, 1000, 1500 and 2000 ppm and those of CO are set at 50, 100, 150 and 200 ppm, respectively. Binary mixtures are produced by respectively mixing CH_4_ at four concentrations and CO at 50, 100 and 150 ppm. Responses of the same composition with different concentrations of gases in the sensor array were considered as one category. Therefore, the classification purpose is to identify three kinds of gases, which are pure CO, pure CH_4_ and mixtures of CO and CH_4_.

**Figure 3:**
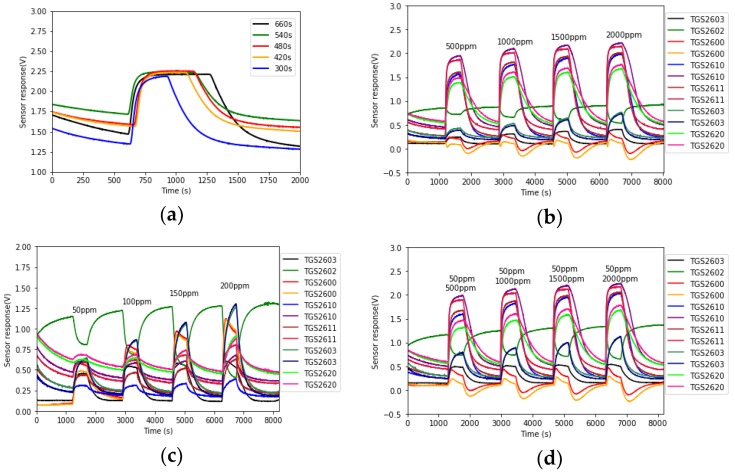
(**a**) Response curves of TGS2603 to CO at 50 ppm concentration at different injecting times; (**b**) Response of 12 sensors to CH_4_ at four concentrations; (**c**) Response of 12 sensors to CO at four concentrations; (**d**) Response of 12 sensors to gas mixtures (50 ppm CO + 500~2000 ppm CH_4_).

For each analyte test, a process of zero air cleansing was performed first for the purpose of cleaning the chamber and stabilizing the gas sensor baseline. This period is set at least at 20 min based on the experiment results. Then the analyte was injected into the chamber. The sensors’ response time is around 30–120 s, and the recovery time is a bit longer. An experiment on the injection time was performed. The CO at 50 ppm concentration was injected into the chamber for 660, 540, 480, 420 and 300 s, respectively. Figure 3a shows the response curves of TGS2603 for these periods. It can be seen that the sensor response was stable during all these periods. Hence as long as the injection time is longer than the sensors’ response time, the sensor response curves could reach a stable point. In the following experiments, the injection time was settled at 480 s.

According to the test process, each concentration of each analyte was measured five times repeatedly. A total of 100 sets of raw data were obtained. The 12 sensor response signals were acquired by the DAQ at a sampling frequency of 1Hz. Some typical measured raw data are shown in Figure 3b–d.

## 3. The Structure of CNN

### 3.1. The LeNet-5 Structure

**Figure 4:**
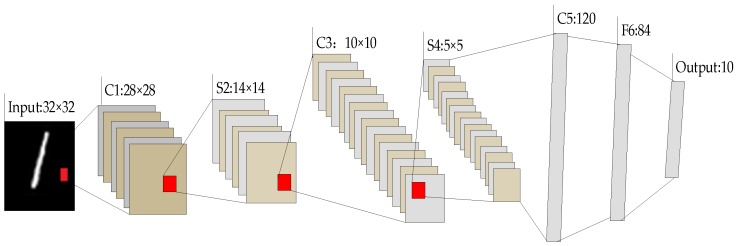
The LeNet-5 structure proposed by Yann LeCun [13].

LeNet-5 is a gradient-based learning CNN structure and first successfully applied in hand-written digital character recognition [17]. The typical LeNet-5 structure diagram is shown in Figure 4. Its input layer is a hand-written digital picture of 0~9 with a size of 32 × 32, and its output layer has 10 nodes corresponding to numbers of 0~9. In addition to the input and output layers, generally LeNet-5 includes six layers, which are three convolutional layers, two pooling layers, and one fully connected layer. The size of convolutional core is set to 5 × 5 in the convolutional layer and the core in the pooling layer is set to 2 × 2. The full connection layer reduces the number of neurons from 120 to 84 to reduce parameter training.

### 3.2. Convolutional Layer

The Convolutional Layer is mainly adopted to perform the feature extraction process. Each layer has a number of convolutional kernels. The input matrix is convolved with the convolution kernel at this layer. Suppose the input matrix is $X=\left\{x_{i,j}|i=1,2,\ldots I,j=1,2,\ldots J\right\}$, I=32 and J=32 in Figure 4. For gas data, *I* is the number of sensors, and *J* is the number of gas data in response. The convolution kernel is denoted as $W=\left\{w_{m,n}|m=0,1,\ldots F-1,n=0,1,\ldots F-1\right\}$, where F denotes the size (i.e., width or height) of the convolutional kernel, which are equal. In Figure 4, F equals to 5. The expression of the convolutional layer is shown in (1):

(1) $$a_{i,j}={\left\{f(\sum_{m=0}^{F-1}\sum_{n=0}^{F-1}w_{m,n}x_{i+m,j+n}+b)\right\}}_{i=1,2,\ldots ,I;j=1,2,\ldots ,J}$$

where $a_{i,j}$ represents the output after convolution, b denotes the offset term for each convolution and f(•) denotes the activation function.

### 3.3. Activation Functions

Generally, there are five widely used activation functions, which are Sigmoid, Tanh, ReLU, Softplus and Gaussian [21]. Sigmoid, Tanh and Gaussian are generally saturating nonlinear functions, shown in Equations (2)–(4), respectively. They are mostly chosen as the activation functions in traditional CNNs:

(2) $$f(x)=\frac{1}{1+e^{-x}}$$

(3) $$f(x)=\frac{e^{x}-e^{-x}}{e^{x}+e^{-x}}$$

(4) $$f(x)=e^{-x^{2}}$$

Currently, unsaturated nonlinear functions are often used as activation functions in CNN structures. The most commonly used functions are ReLU functions and Softplus functions, which are shown in Equations (5) and (6), respectively:

(5) f(x)=max(0,x)

(6) $$f(x)=\ln(1+e^{x})$$

**Figure 5:**
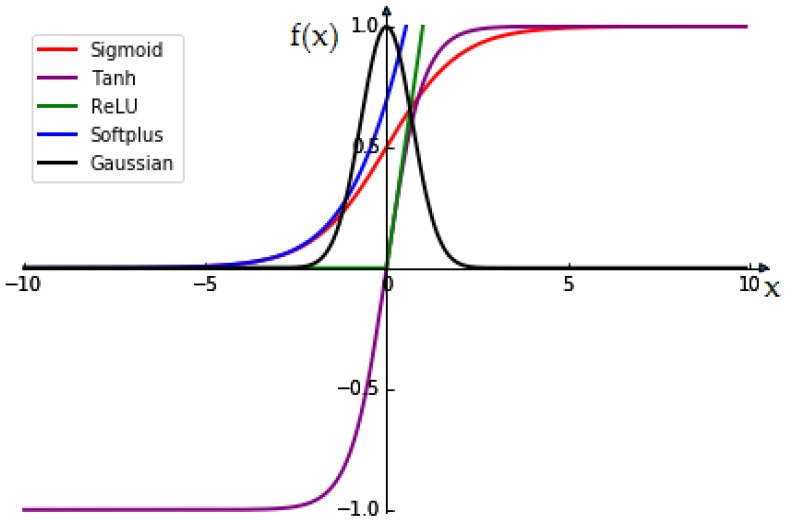
Activated functions.

The five activation functions are shown in Figure 5. It can be seen that the output space of the Sigmoid and Gaussian function are at (0, 1), and the output space of the Tanh function is at (−1, 1). When the input is too large, the output of the Sigmoid function and the Tanh function tends to 1 and remains stable, but the Gaussian function tends to 0 as the input increases.

When the input is too small, the output of the Sigmoid function and the Gaussian function tends to 0 and remains stable, but the Tanh function tends to −1 as the input decreases. The output of the three activation functions may be close to smooth. Therefore, their gradient is very close to zero, which is not conducive to updating weight. From the above analysis, it can be concluded that there is a problem of gradient explosion and gradient disappearance in saturating nonlinear functions. Different activation functions in CNN are discussed in Ref. [22]. It is found through experiments that the unsaturated nonlinear function can not only solve those problems, but also accelerate the convergence speed and improve the performance of CNN [23,24].

It can be seen from the green and black lines in Figure 5 that the ReLU function and the Gaussian function have no gradient saturation problem when the input is positive, and they are much faster than saturating nonlinear functions. In Ref. [21], the ReLU function is also adopted. Hence the ReLU function is chosen as the activation function in our CNN.

### 3.4. Pooling Layer

The purpose of the pooling layer is to perform a feature selection process to reduce the data dimensions while conserving the main characteristics of the data. Maximum pooling, mean pooling and randomly pooling are generally used approaches, which extract the points with the largest value, mean value and random values in the local accepted domain [25]. In the LeNet-5 structure, the pool size of 2 × 2 is used, which means that the input feature matrix is reduced by two times in two dimensions. The expression of the pooling layer is shown in Equation (7), where pool(•) represents the maximum pooling operation. Generally, the output of lth layer is denoted as $a_{n}^{l}$ and $a_{n}^{l-1}$ denotes the output of former layer, where *n* is corresponding to the *n*th sample:

(7) $$a_{n}^{l}=pool(a_{n}^{l-1})$$

In the designed CNN, the combination of convolution, ReLU and pooling plays the role of feature extraction, which could be used equivalently to feature extraction in the traditional gas identification. But the CNN process can not only replace the tedious feature design in the gas identification, but also reduce the network parameters with the design idea of partial sensory field and weight sharing.

### 3.5. Fully Connected Layer

The fully connected layer is generally the last layer in the structure of CNN. Each neuron uses the ReLU activation function, which is fully linked to the neurons of the previous layer. The fully connected layer can integrate local information, which has the ability of discriminating classes [26], and the neuron output is passed to the output layer. Therefore, the fully connected layer has some role of conventional classifiers. If the lth layer is the fully connected layer, the output of this layer will be composed by Equation (8), where $w^{l}$ denotes convolutional kernel and $b^{l}$ denotes the offset term:

(8) $$a_{n}^{l}=f(w^{l}\cdot a_{n}^{l-1}+b^{l})$$

### 3.6. Output Layer

**Figure 6:**
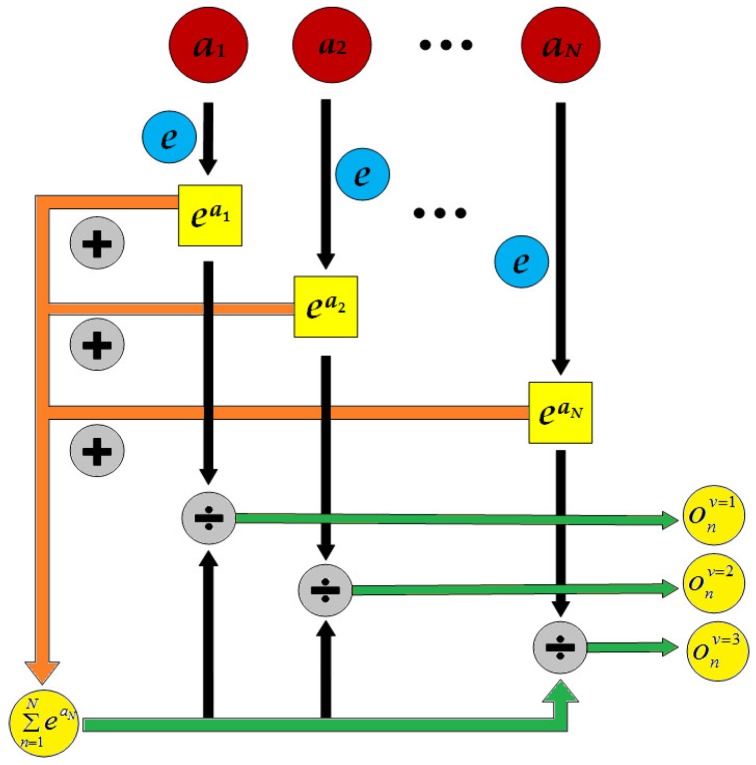
The classification process of softmax.

Output layer is also called the softmax layer, which is represented by Equation (9). The softmax function is mainly used in the multiple classification process, which maps the output of the fully connected layer to (0, 1). Each output corresponds to the probability of classification, and their cumulative sum is 1. Finally, the classification of the maximum probability is selected as the output. The process of the softmax function is shown in Figure 6:

(9) $$a_{n}^{L}=o_{n}^{v}=\mathrm{softmax}(w^{L}\cdot a_{n}^{L-1}+b^{L})$$

The probability of different classification categories obtained by softmax is denoted by $o_{n}^{v}(v=1,2,3,\ldots V;n=1,2,3,\ldots N)$, which indicates the output probability of the *n*th sample for *v* different classified categories. If $t_{n}^{v}$ represents the expected output probability of the *n*th sample in *v* different classification categories, the error formula *E_n_* corresponding to the *n*th sample will be obtained by Equation (10):

(10) $$E_{n}=\frac{1}{2}\sum_{v=1}^{V}{\left\|t_{n}^{v}-o_{n}^{v}\right\|}_{2}^{2}$$

and the global error of *N* samples could be obtained by Equation (11):

(11) $$E=\sum_{n=1}^{N}E_{n}$$

Based on the above analyses, the fully connected layer and output layer might be equivalent to the classifiers in traditional gas identification. When the CNN network is trained by small data, the training results are prone to over-fitting. In order to avoid over-fitting, the dropout technique prevents some random neurons from making forward propagation of CNN. Therefore, the learning of neurons has more robust features. At present, most research of CNN adopts ReLU and dropout technology, which has achieved good classification performance [27,28].

## 4. The Algorithm of CNN

The general algorithm contains two sub-algorithms, which are the forward propagation and the backward propagation one.

### 4.1. The Forward Propagation Algorithm

The forward propagation algorithm is presented in Algorithm 1. The output of the forward algorithm is *E*, which represents the error between the expected output and the actual output. In training set, *x_n_* represents the input of data matrix and *y_n_* represents the label of data.


**Algorithm 1 The forward propagation algorithm of CNN**
1 //process of the forward propagation2 **Input:** training set $D={\left\{\left(x_{n},y_{n}\right)\right\}}_{n=1}^{N}$; the number of CNN layers is L, each layer denoted as $h_{l}$; $a_{n}^{l}$ represents the nth input sample corresponding to the output of layer l; expected output $t_{n}^{v}$.3 **Process:**4 **Initialization:** Initialize all layers of convolutional kernel $w^{l}$ and offset term $b^{l}$.5 for all $(x_{n},y_{n})\in D$ do6  for (l=1;l≤L;l+1) do7   if ($h_{l}$ is the convolutional layer) then8     for (all $a_{n}^{l}$) do9     get $a_{n}^{l}$ according to (1)10    end11   end12   if ($h_{l}$ is the pooling layer) then13    for (all $a_{n}^{l}$) do14     get $a_{n}^{l}$ according to (7)15    end16   end17   if ($h_{l}$ is fully connected layer)18    for(all $a_{n}^{l}$) do19     get $a_{n}^{l}$ according to (8)20    end21   end22   if ($h_{L}$ is output layer) then23    get $a_{n}^{L}$ or $o_{n}^{v}$ according to (9)24   end25  end26 end27 **Output:** Calculate the error E of the output layer by the loss function, according to (11).

### 4.2. The Reverse Propagation of CNN

Let $z^{L}=w^{L}\cdot a_{n}^{L-1}+b^{L}$, $\delta^{L}=\frac{\partial E}{\partial z^{l}}$, and $\delta^{l}$ of the previous hidden layers can then be obtained by the reverse propagation. The reverse propagation algorithm of CNN is summarized in Algorithm 2, which is mainly to update the weight *w* and offset *b* of the convolutional layers and the fully connected layers.


**Algorithm 2 The reverse propagation algorithm of CNN**
1  //process of the reverse propagation2 **Input:** The error E of the output layer calculated by the loss function, the learning rate γ,γ∈(0,1).3  **Process:**4  for (l=L;l≥1;l=l−1) do5    if ($\delta_{n}^{l+1}$ is fully connected layer) then6    get $\delta_{n}^{l}$ according to $\delta_{n}^{l}={\left(w^{l+1}\right)}^{T}\delta_{n}^{l+1}⊙f^{\prime }(w^{l}a_{n}^{l-1}+b^{l})$7    end8    if ($\delta_{n}^{l+1}$ is the convolutional layer) then9    get $\delta_{n}^{l}$ according to $\delta_{n}^{l}=\delta_{n}^{l+1}\frac{\partial [f(z^{l})\times w^{l+1}+b^{l+1}]}{\partial z^{l}}\times rot180(w^{l+1})⊙f^{\prime }(w^{l}\times a_{n}^{l}+b^{l})$10   end11   if ($\delta_{n}^{l+1}$ is the pooling layer) then12    get $\delta_{n}^{l}$ according to $\delta_{n}^{l}=upsample(\delta_{n}^{l+1})⊙f^{\prime }(pool(a_{n}^{l}))$13   end14  end15  for (l=2;l≤L;l=l+1) do16   if($h^{l}$ is fully connected layer) then17    $w^{l}=w^{l}-\gamma \sum_{n=1}^{N}\delta_{n}^{l}{\left(a_{n}^{l-1}\right)}^{T}$18    $b^{l}=b^{l}-\gamma \sum_{n=1}^{N}\delta_{n}^{l}$19   end20   if($h^{l}$ is the convolutional layer) then21    $w^{l}=w^{l}-\gamma \sum_{n=1}^{N}\delta_{n}^{l}\times rot180(a_{n}^{l-1})$22    $b^{l}=b^{l}-\gamma \sum_{n=1}^{N}\sum_{u,v}{\left(\delta_{n}^{l}\right)}_{u,v}$23   end24  end25 **Output:** Updated values for w and b.

## 5. Design of Gas Recognition Algorithm Based on CNN

### 5.1. Gas Data Preprocessing

Based on the data measurement process in Section 2.3, the sensor array was exposed to the test analyte for a specified period and response curves were sampled at a rate of 1 Hz. Hence the response curves at the analyte injection time contain the sensor response information. This part of the array curves was extracted as the raw data. In our experiments, the injection time was set at 8 min and 12 sensors were used, which means that each raw data has a size of 480 × 12. Suppose *X* represents the raw response matrix, and $X=\left\{x_{i,j}\right\}$, where i=1,2,…,480 represents the sample time and j=1, 2, …,12 represents 12 sensors. It can be seen from Figure 3 that gas sensor response curves vary slowly when injecting the target gases. Therefore, we can use less data to represent the information. 

To further reduce the dimensions of the input data, the sensor response curves are resampled by M=480/N,M≥12, where N is the sampling interval. If *N* takes 40, 30, 20 and 10, the data is then downsampled with sizes of 12 × 12, 16 × 12, 24 × 12 and 48 × 12. If the original data size is not 480, downsampling can also be performed with other intervals. Here the uniform downsampling is performed. 

**Figure 7:**
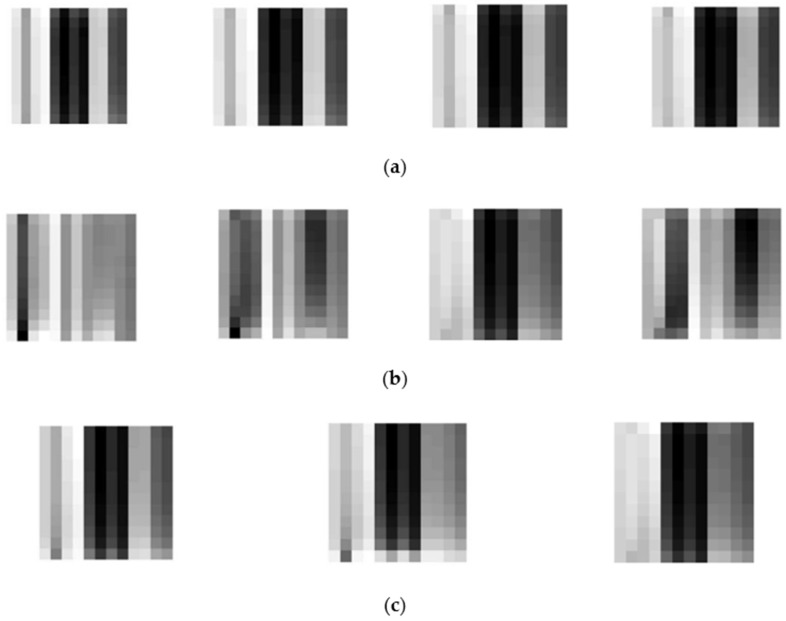
(**a**) Patterns of CH_4_ data matrices with size 12 × 12 (500, 1000, 1500, 2000 pm); (**b**) Patterns of CO data matrices with size 12 × 12 (50, 100, 150, 200 pm); (**c**) Patterns of mixture data matrices with size 12 × 12 (500 ppm CH_4_ + 50 ppm CO, 500 ppm CH_4_ + 100 ppm CO, 500 ppm CH_4_ + 150 ppm CO).

The downsampled data matrix is then normalized to the space of (0, 1) and rescaled to the space of (0, 255) by Equation (12):

(12) $${x\_rescale}_{i,j}=\frac{x_{i,j}-\min(x)}{\max(x)-\min(x)}\times 255$$

where min(*x*) and max(*x*) are the minimum value and maximum value of *X* for each sensor *j*. Then the rescaled data are transformed to the integers and can be shown as grayscale patterns. Figure 7 shows some typical patterns of CH_4_, CO and gas mixtures. Each preprocessed grayscale pattern represents the information of sensor array corresponding to the test analyte. 

### 5.2. The Dataset Augmentation

Deep learning methods usually need large amounts of training data, which is quite a challenge for EN detection. As we can see, the time for each test was 28 min in our case. Before each test, chamber cleansing also needs time. The gas sensors will need a preheating time of 3 days at least if they are not used for a long time. Therefore, the data measurements of ENs are quite time-consuming. Hence data augmentation techniques were considered.

For small sampling data set, data augmentation techniques such as cropping, panning, scaling and rotation are usually used to augment the data size. In our case, translation and cropping were performed on the 100 sets of raw data. Another reason for considering data translation is that gas sensor response curves vary slowly when injecting the target gases and downsampling has been used to reduce the data. Therefore data translation will not change the gas information clearly but some baseline drift could be added into the augmented dataset. In our case, *X* is translated with a step of 2η(η∈[0,9]), shown in (13), then 100 × 10 = 1000 data sets $X_{\eta }$ are obtained:

(13) $$X_{\eta }=\left\{x_{i+2\eta ,j}\right\}$$

### 5.3. The Gas Recognition Algorithm Based on CNN

In Algorithm 3, *E* represents the error; *e* represents the set error value; the *k* represents the number of iterations. If the error *E* is greater than the set error *e* by the forward propagation Algorithm 1, the weight *w* and the threshold *b* are updated and the forward propagation algorithm is returned to calculate a new error *E*. If E≤e, the iteration is stopped and the weight *w* and threshold *b* are output.


**Algorithm 3 The gas recognition algorithm based on CNN**
1   **Input:**
*E* represents the error; *e* represents the set error value; *k* represents the number of iterations and *K* represents the maximum batch; $D_{total}$ represents all data sets.2   **Process:**   **//training the LeNet-5**3   for $D_{total}$ do4    One-Hot encoding and data set partition5    *training dataset* and *testing dataset* are obtained6   Begin of training time7   for all *training dataset* do8    for (k=0;k=k+1;k<K) do9     Algorithm 110    if E>e then do11     Algorithm 2 and return to step 812    else13     break;14    end15   end16  end17  End of training time18  store all w and all b   **// test the LeNet-5**19  Load all w and all b to the LeNet-5 then do20  Begin of test time21  for all *testing dataset* do22   Algorithm 1 which is forward propagation23  end24  End of test time25  Calculate the accuracy26  **Output:** training time, test time and accuracy.

## 6. Results and Analysis

The CNN for ENs is trained by the preprocessed data, and the parameters of Gas CNN are studied by detailed experiments. In the training process, 20% of the data is randomly taken out as the verification data set. Therefore, the number of training data sets is 800 and the number of testing data sets is 200.

### 6.1. Influence of the Number of Convolutional Kernels of Gas CNN

**Figure 8:**
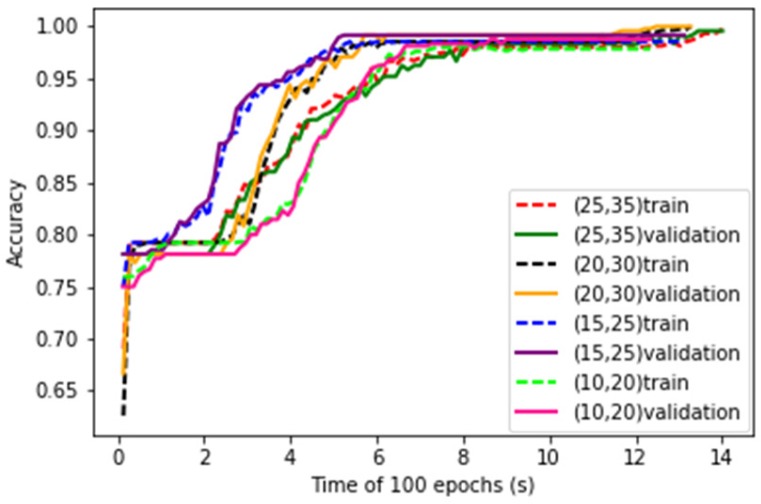
Training and validation curves of LeNet-5 with different number of convolution kernels.

The numbers of convolutional kernels are key parameters of LeNet-5 structure. Four kinds of parameter combinations are studied. The convergence curves of training process of the LeNet-5 at the four combinations are shown in Figure 8. It can be seen that with the increase of number of kernels, the convergence speed of the learning curves decreases.

After training, the test data are put into the LeNet-5. The test accuracy and running time of the LeNet-5 at four combinations of convolution kernels are obtained and shown in Table 2. It can be seen that as the number of convolution kernels increases, the accuracy rate increases during the early stage and then decreases, but the running time has been increasing.

It is conceivable that the greater the number of convolution kernels, the more amount of each convolution process will increase, so the curve fitting time will become longer. As each time the feature is extracted from the data becomes more specific, the accuracy will also increase. Trading off the accuracy and the training time, the number of convolutional kernels of C1 and C3 are set to 20 and 30 respectively for the following experiments.

### 6.2. Influence of the Size of Convolutional Kernels of Gas CNN

**Figure 9:**
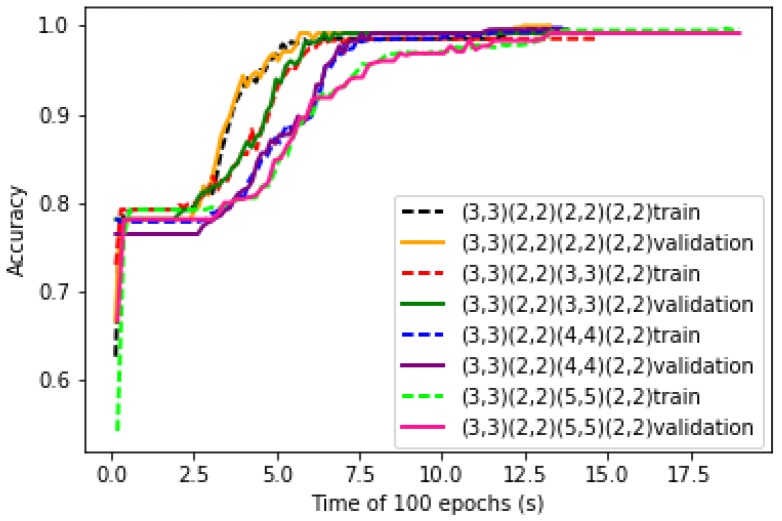
Training and validation curves of LeNet-5 with different sizes of convolutional kernels.

The sizes of convolutional kernels are also key parameters of the LeNet-5 structure. Four different sizes of convolutional kernels in C3 are studied in the structure of CNN. The convergence curves of the training process of four different sizes of convolutional kernels are shown in Figure 9. It can be seen that as the size of the convolutional kernel increases, the convergence rate of the learning curve decreases.

For the convolutional layer and the pooling layer, there are two padding ways to fill the data which are the ‘Valid’ padding and the ‘Same’ padding. The ‘Same’ padding method is to enhance the extraction of edged data features, while its input data and output data are equal in size. The disadvantage is that its convolutional kernel size only can be odd number. But for the ‘Valid’ padding, the size of the convolutional kernel can be even.

Based on the LeNet-5 structure and the input characteristic of gas data, four different sizes of convolutional kernels are studied and the ‘Valid’ padding approach is adopted. The experimental results are shown in Table 3. The time becomes longer as the size of convolutional kernel becomes larger. The accuracy of the 2 × 2 convolutional kernel is the highest. It shows that it has a more comprehensive extraction function. When the output of the 3 × 3 convolutional kernel is used as the input to the pooling layer, the outermost features are lost and the accuracy is the lowest. Therefore, a 2 × 2 convolutional kernel is most optimal in the C3 layer with the ‘Valid’ padding approach.

### 6.3. Influence of Size of Inputs

**Figure 10:**
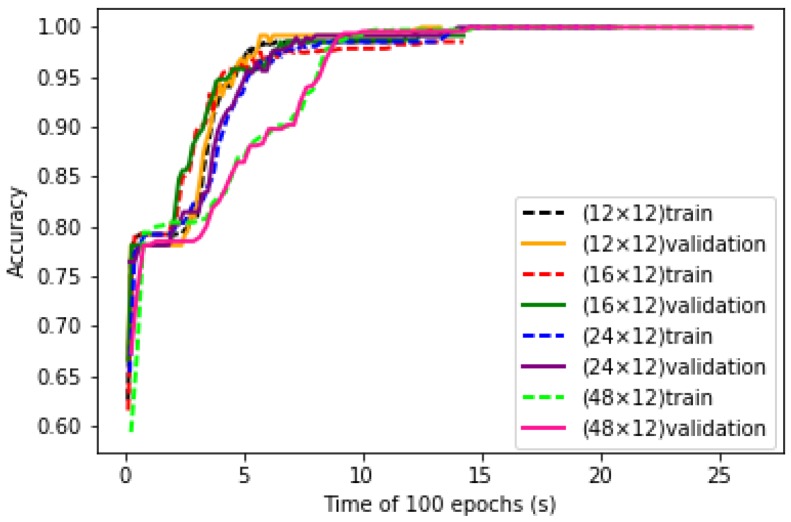
Training and validation curves of LeNet-5 with different sizes of inputs.

Four sizes of input matrix are studied. These data sets are used to train the LeNet-5 structure. The convergence curves of training process of LeNet-5 with four sizes of inputs are shown in Figure 10. It can be seen that with the increase of input matrix sizes, the convergence speed of LeNet-5 increases, which means that larger size of data input contains more information. However, with smaller size of input, after enough time of generalization of the structure, satisfied accuracy could also be reached.

Test data sets are taken as the input of LeNet-5, the accuracy and running time are shown in Table 4. It can be seen that with the increase of input data size, the accuracy increases, while the running time increases greatly. It is conceivable that the input data does not lose important features and achieve the desired minimum. Although the accuracy rate will be reduced, the running time will be greatly reduced. Hence, the suitable size of the selected data is set at 12 × 12.

### 6.4. Improved LeNet-5 Structure for ENs

**Figure 11:**
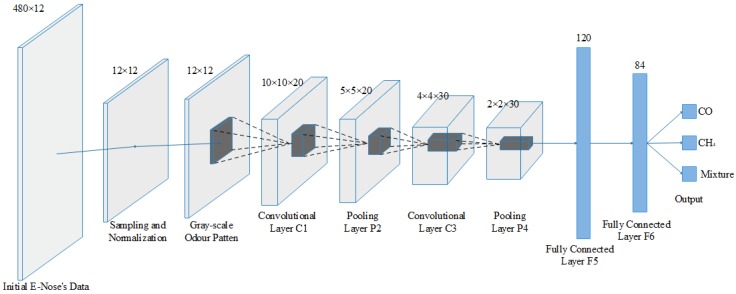
Improved LeNet-5 structure for ENs.

In order to adapt to the practical EN in our case, the improved structure and design of LeNet-5 are shown in Figure 11. The input layer is the gas sensor feature matrix with size of 12 × 12. C1 and C3 are the convolutional layers with kernel size of 3 × 3 and 2 × 2, respectively, and their numbers of convolutional kernels are 20 and 30, respectively. The outputs of C1 and C3 after convolution are 20 matrices with size of 10 × 10 and 30 matrices with size of 4 × 4, respectively. S2 and S4 are pooling layers with the same kernel size of 2 × 2. The dropout coefficient is 0.3, hence the number of neurons is 120 in the F5 layer and 84 in the F6 layer. The output layer contains three neurons based on the targets, corresponding to three target categories of CH_4_, CO and their mixtures, respectively.

Each layer in the designed CNN structure has parameters that require training. In each layer of the network structure, the parameters are shown in Table 5. And the number of neurons is shown in Equation (14), where *filter_w_* and *filter_h_* represent the width and the height of the convolutional kernel, respectively. *number_filters_* represents the number of convolutional kernels.

(14) $$\text{No. of Neurons}=({filter}_{w}\times {filter}_{h}+1)\times {num}_{filters}$$

### 6.5. Comparison with Other Shallow Models

**Figure 12:**
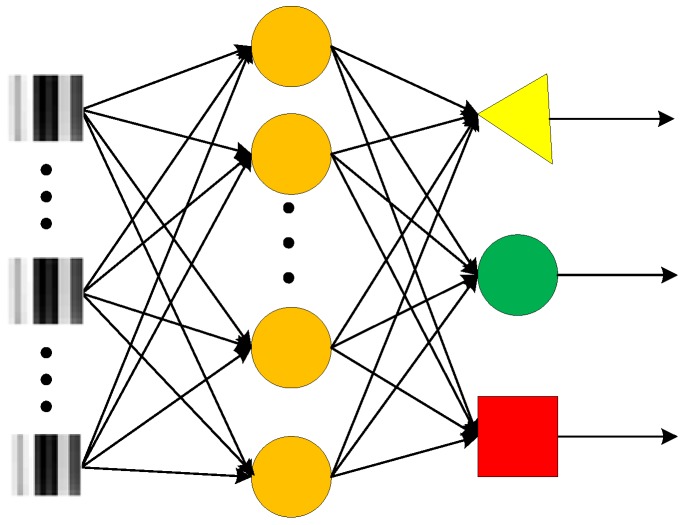
The structure of the MLP.

To verify the performance of the improved Gas CNN structure, the same processes are performed on the generally used shallow models MLP, PNN and SVM. MLP is a generally used feed- forward artificial neural network model in gas recognition. For effective comparison, two kinds of MLP NN structures are set while their numbers of hidden layers were set to 50 and 10, respectively. Figure 12 shows the structure of MLP NN with 10 hidden neurons. In addition to MLP, PNN and SVM are also used as comparison algorithms. All the shallow models are processed with the same input and the ReLU activation function is adopted, which is the same as the Gas LeNet-5.

Comparison results are shown in Table 6. It can be seen that higher accuracy is obtained by improved LeNet-5, and the training time of LeNet-5 is the longest. However, after training the test time of the improved LeNet-5 is at the same level with the MLP, PNN and SVM. This infers that higher accuracy can be obtained by deep CNN models while the shallow models that are commonly used have almost the same recognition time.

### 6.6. Influence of Data Augmentation

All the above analyses are based on a 10-times augmented dataset by translation of the original sensor curves. In order to measure the influence of the data augmentation, 10 percent of original data set was randomly selected out and their translated sampling data were used as the test set, and the remaining data and their translated sampling data were used as the training set. The performances of the models were measured and shown in Table 7. Compared with Table 6, it can be seen that the accuracy of all the models decreases, because none of the information of test set had been put into the training part. The influence of the data augmentation is the lowest. But the improved LeNet-5 still has the highest accuracy compared with other shallow models.

## 7. Conclusions

The current research aim was to identify CH_4_, CO and gas mixtures of CH_4_ and CO by means of electronic nose and LeNet-5 in CNN. Firstly, according to the characteristics of gas data and CNN structure, an algorithm suitable for gas identification is designed. Then, we discussed the parameters of CNN structure, including the size of input data, the number of convolution kernels and the size of convolution kernels. Finally, considering the accuracy and computation time, the LeNet-5 for ENs is developed.

After parameter setting, a complete improved LeNet-5 structure is obtained for gas identification. In order to avoid overfitting and obtain more reliable statistical results, we extend the gas data by means of translation. The matrix data is transformed into gray image to make the difference between different kinds of data more considerable. Based on the improved gas LeNet-5, the test accuracy of three categories of gases could reach 99.67% with the fully augmented dataset and 98.67% with unused original dataset. Compared with general MLPs, PNN and SVM, the improved gas CNN obtained higher classification accuracy, which proves the effectiveness of the structure and algorithm, while requiring a same time cost level.

## Figures and Tables

**Table 1:** Metal oxide gas sensors in the designed array.

Sensor Part No.	TGS2603	TGS2602	TGS2600	TGS2610	TGS2611	TGS2620
**Channel**	0, 8, 9	1	2, 3	4, 5	6, 7	10, 11

**Table 2:** Comparison of results with different number of convolutional kernels.

Methods	Parameters	Accuracy	Time(s)
**Number of kernels of (C1, C3)**	(10, 20)	97.83%	0.01540
	(15, 25)	98.67%	0.01546
	(20, 30)	99.67%	0.01553
	(25, 35)	99.50%	0.01568

**Table 3:** Comparison of results with different size of convolutional kernels.

Methods	Parameters	Accuracy	Time(s)
**Size of C3 kernels**	2 × 2	99.67%	0.01553
	3 × 3	98.67%	0.01557
	4 × 4	99.59%	0.01607
	5 × 5	99.00%	0.01591

**Table 4:** Analysis of results with different data sizes.

Methods	Parameters	Accuracy	Time(s)
**Input data size**	12 × 12	99.67%	0.01553
	16 × 12	98.67%	0.01559
	24 × 12	99.71%	0.01563
	48 × 12	99.73%	0.01615

**Table 5:** The parameters of improve LeNet-5 structure.

Layer	Activation Shape	Activation Size	Parameters	No. of Neurons
Input	(12, 12, 1)	144		0
CONV1	(10, 10, 20)	2000	$w_{Conv1}$	200
POOL2	(5, 5, 20)	500		0
CONV3	(4, 4, 30)	480	$w_{Conv3}$	150
POOL4	(2, 2, 30)	120		0
FC5	(120, 1)	120	$w_{FC5}$	14,401
FC6	(84, 1)	84	$w_{FC6}$	10,081
Softmax	(3, 1)	3	$w_{\mathrm{Softmax}}$	253

**Table 6:** Comparison of performances with shallow models.

Model	Accuracy	Train Time(s)	Test Time(s)
MLP (10)	95.55%	6.131	0.01505
MLP (50)	95.55%	9.703	0.01506
PNN	93.33%	1.560	0.01497
SVM	85.70%	1.156	0.02040
Improved LeNet-5	99.67%	12.730	0.01553

**Table 7:** Comparison of performances with shallow models tested by untrained original data.

Model	Accuracy	Train Time(s)	Test Time(s)
MLP (10)	93.00%	6.503	0.01505
MLP (50)	95.00%	10.083	0.01506
PNN	90.70%	1.632	0.01512
SVM	82.20%	2.207	0.01702
Improved LeNet-5	98.67%	16.146	0.01553
